# Structural and antigenic characterization of *Babesia Bovis* HAP2 domains

**DOI:** 10.1038/s41598-025-91359-4

**Published:** 2025-03-05

**Authors:** S. M. Raihan Rahman, Heba F. Alzan, Jacob M. Laughery, Reginaldo G. Bastos, Massaro W. Ueti, Carlos E. Suarez

**Affiliations:** 1 Department of Veterinary Microbiology and Pathology, College of Veterinary Medicine, Washington State University, Pullman, WA USA; 2 Parasitology and Animal Diseases Department, National Research Center, Dokki, Giza, Egypt; 3 Animal Disease Research Unit, United States Department of Agriculture - Agricultural Research Service, Pullman, WA USA

**Keywords:** Bovine babesiosis, *Babesia Bovis*, HAP2, HAP2 domains, Transmission-blocking vaccine, Biotechnology, Computational biology and bioinformatics, Immunology, Microbiology, Molecular biology, Zoology, Diseases

## Abstract

The tick-borne apicomplexan parasite *Babesia bovis* causes bovine babesiosis which leads to enormous food and economic losses around the world. The existing resources to manage this disease are limited and have pitfalls, therefore, introduction of new strategies is urgently needed. *B. bovis* reproduces sexually in the midgut of its tick vector. HAP2, a well conserved ancient protein, plays a crucial role in the gamete fusion of this parasite and is a strong candidate for developing transmission-blocking vaccines. We previously demonstrated that immunization of cattle with full size *B. bovis* HAP2 blocks transmission of the parasite by *Rhipicephalus microplus*. Understanding the conserved structural features and antigenicity of HAP2 protein and its domains will facilitate developing effective methods to control pathogen transmission. In this study, we analyzed and compared AlphaFold2-predicted 3D structure of *B. bovis* HAP2 with the well-characterized crystal structures of HAP2 of *Chlamydomonas reinhardtii* and *Arabidopsis thaliana*. The comparisons and structural analysis resulted in the definition of three domains’ sequences, fusion loops, and disulfide bonds in the *B. bovis* HAP2. In addition, recombinant versions of each three predicted HAP2 domains were recognized by antibodies from HAP2 immunized and transmission-protected cattle, confirming their antigenicity. Remarkably, domain II was highly recognized compared to the other two domains. This study introduces new directions in designing novel functional assays and improved vaccine design through targeting the HAP2 protein.

**Supplementary Information:**

The online version contains supplementary material available at 10.1038/s41598-025-91359-4.

## Introduction

Bovine babesiosis is a tick-borne disease that is caused by apicomplexan parasites of the genus *Babesia*, mainly *Babesia bovis*,* B. bigemina*, and *B. divergens.* Bovine babesiosis causes significant economic losses worldwide, ranging annually from US$ 573.6 million to 3.24 billion. While *B. bovis* is the predominant and most virulent causative agent of this disease, *B. divergens* is also a zoonotic organism, responsible for human babesiosis, a disease that can also be caused by the sensu lato *B. microti*. The most important vector of *B. bovis* is the *Rhipicephalus microplus* tick^1–4^. More than 500 million cattle around the world are susceptible to this disease. In the US, eradication of bovine babesiosis through the elimination of *R. microplus* saves $3 billion annually, but the risk of its reappearance is still in effect^1–5^. Multiple control strategies have been applied to limit this disease, but unfortunately, they all have drawbacks. This include the development of resistance against most if not all acaricides used to clear cattle infestations by ticks, high costs of live vaccine production and transportation, the need for a cold chain, and the risk for reversion to virulence, among others^6–9^. Various efforts are undergoing to develop vaccines targeting different life stages of *B. bovis*, including blood stage and transmission-blocking vaccines (TBV), which are designed to target the blood stage and sexual stage development, respectively^6,10–17^.

*B. bovis* has a complex dixenic life cycle that includes asexual replication in its vertebrate hosts, and sexual reproduction in the gut of its tick vector. The parasite is acquired with a blood meal by the tick from the vertebrate host and then, gametocytes are formed in the lumen of tick midgut differentiating in male and female gametes. Gametes then fuse to form zygotes which develop into kinetes that invade different regions of the tick, including the ovaries and consequently the eggs of the next progeny, resulting in transovarial transmission^3,6,18–20^. Though the events occurring during the development of sexual reproduction of *B. bovis* are not fully defined yet, previous studies demonstrated the expression of some proteins that play vital roles in this stage. These include some members of the AP2 family as well as members of the CCP and 6cys families, and HAP2 (Hapless 2)^3,17,21–23^. In addition, *pka* (cyclic adenosine 3’,5’-monophosphate (cyclic AMP)-dependent protein kinase cAMPDPK), *hap2*, *α-tubulin II*, *znfp2* (zinc finger C3H1 protein2) were identified as male gamete specific genes, while, *α-tubulin I*, *trap2-4*(thrombospondin-related anonymous proteins), ABC transporter and *ccp1-3*(LCCL domain-containing proteins)where found specifically expressed in the female gametes^24^. These studies improved our understanding of some mechanisms involved in the development of the sexual stages of *B. bovis* and set the rationale for developing TBVs. Furthermore, at least two previous studies evaluated the transmission blocking ability of the 6cys A & B and HAP2 proteins in the bovine model. While immunization with a vaccine including the two 6cys proteins did not result in the blockade of transmission of the parasite, cattle immunization with HAP2 elicited immune responses that were able to completely block transmission^14,25^.

HAP2 is a membrane-fusion protein conserved among eukaryotic plants, protozoan and metazoan, which is structurally homologous to viral class II fusogen^26–31^. Furthermore, HAP2 was originally identified in the flowering plant *Arabidopsis thaliana* as a male specific protein^32^. Later, it was also identified in *Lilium longiflorum* pollen named as Generative Cell Specific 1(GCS 1)^33^. A previous study demonstrated that a *B. bovis* cell line with a knockout (KO) in the *hap2* gene is unable to develop sexual forms in in vitro induction assays, suggesting an important role of HAP2 in the sexual reproduction of the parasite^3^. Also, HAP2 is deemed as a potential transmission-blocking vaccine candidate in *Plasmodium* species^34–37^.

Several studies have determined that the HAP2/fusion proteins of various microorganisms and viruses have three extracellular structural domains, which were named DI, DII, and DIII. In addition, previous studies in *Plasmodium* demonstrated that the pre-fusion state of HAP2 is a monomer, which forms a trimer during fusion. The loops at the tip of the DII domain are hydrophobic and variable. The number of fusion loops of the HAP2/fusion proteins varies among organisms. It is known that insertion of the loops into the membrane of a target gamete cell initiates the trimerization of the HAP2, and trimer formation starts at the fusion loops of the DII domain. While DI and DII domains play important roles in trimer formation by making the trimeric core, DIII plays a crucial part in fusion through folding back to this trimeric core. The structural maneuver of the domains leads to the formation of a fusion pore, and ultimately, to the fusion of the two gametes^38–47^. It remains unknown whether these structural features are also present in *Babesia* HAP2 proteins, and if the series of events leading to gamete fusion also occur during sexual reproduction of the parasite. Structural analysis of Babesial HAP2/fusion proteins may help our efforts in understanding sexual reproduction processes in this parasite and develop novel strategies targeting functionally relevant domains. Eliciting antibodies against these domains may block the function of this protein^35,36,41,42,46–48^, abrogating gamete fusion.

In this study, we investigated the structural alignment of *B. bovis* HAP2 with the well-characterized HAP2 ectodomains *of C. reinhardtii* and *A. thaliana* to define the presence of three structural domains. Also, we defined the primary structure, expressed recombinant proteins representing each of the domains, and performed antigenicity analysis of the three so-defined HAP2 domains of *B. bovis*. In addition, recombinant versions of each three predicted HAP2 domain were recognized by antibodies in the HAP2 immunized and transmission-protected bovine sera, confirming their antigenicity. Unravelling the domains/regions of *B. bovis* HAP2 may facilitate the design of novel therapeutics by identifying immunologically and functionally important domains/regions involved in gamete fusion of this parasite.

## Results

### Structural analysis of *B. bovis* HAP2

**Fig. 1:**
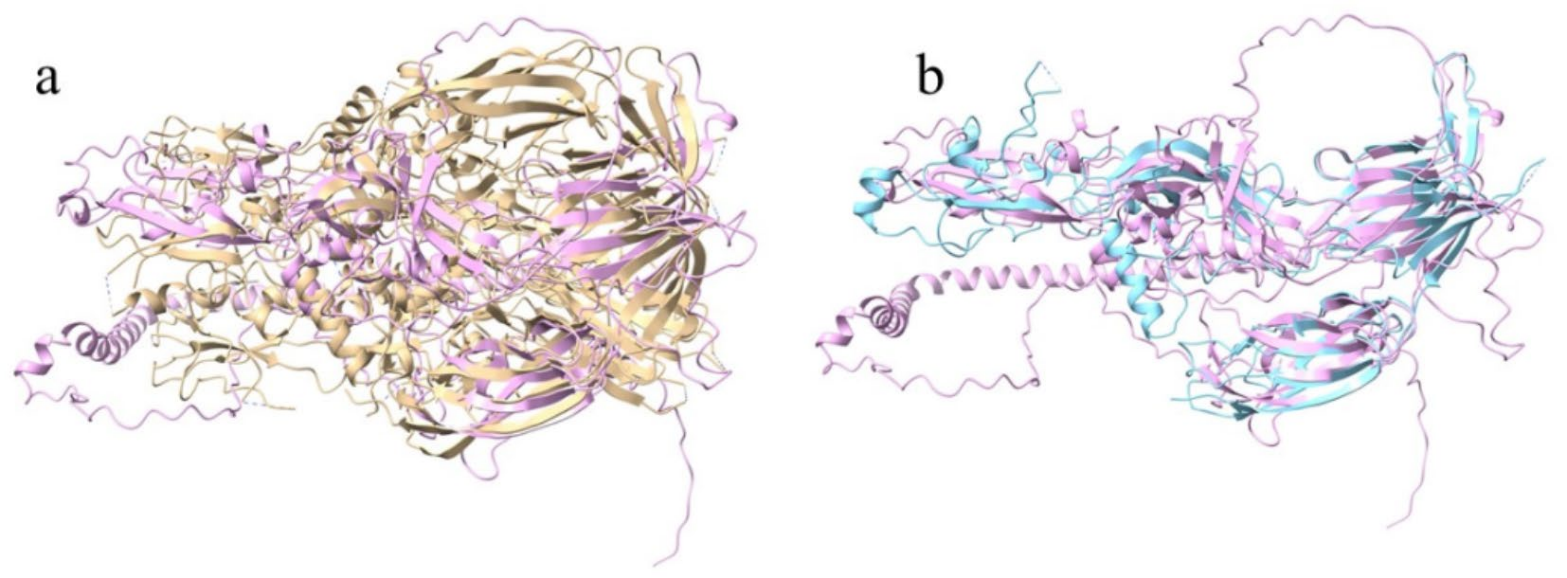
Structure comparisons: (**a**) Superimposition of the HAP2 of *B. bovis* (pink) with the HAP2 of *C. reinhardtii* (gold); (**b**) Superimposition of the HAP2 of *B. bovis* (pink) with the HAP2 of *A. thaliana* (cyan).

**Fig. 2:**
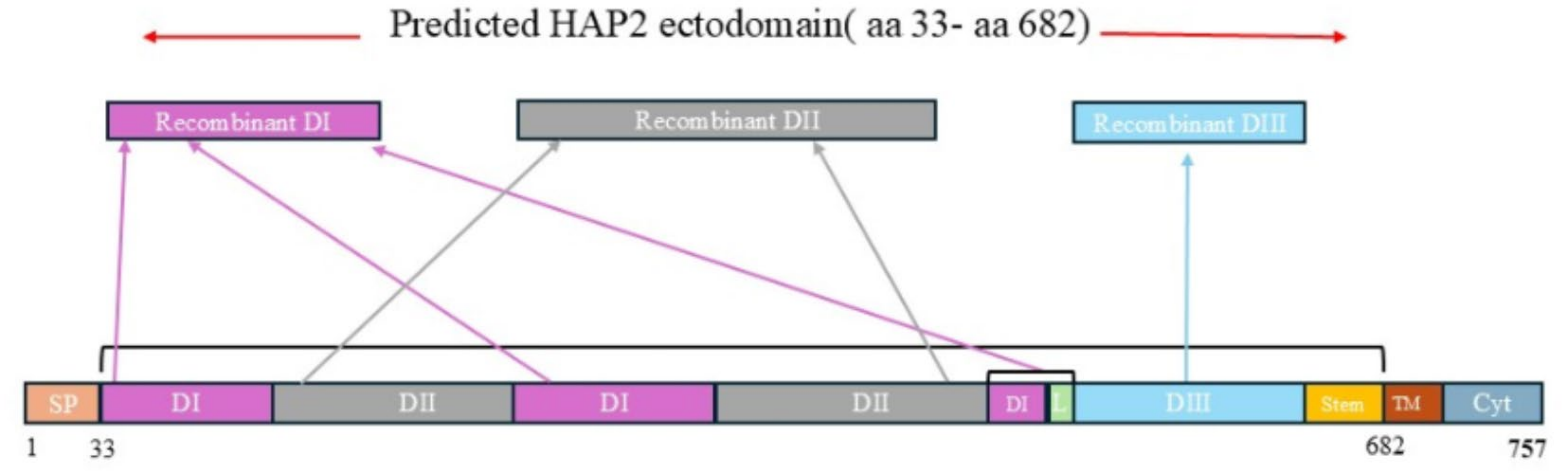
Schematic diagram of *B. bovis* HAP2 primary structure. Signal peptide (SP), Domain I (DI), Domain II (DII), Domain III (DIII), Linker (L) of DI-DIII, Transmembrane region (TM), Cytoplasmic region (Cyt). The recombinant domains DI and DII were designed adding the discontinuous amino acid sequences of each of these domains.

The *B. bovis* HAP2 AlphaFold2 predicted structure was superimposed with the HAP2 structures of *C. reinhardtii* and *A. thaliana* (Fig. 1). The superimposition showed that the root means square deviation (rmsd) value between *B. bovis* HAP2 and *C. reinhardtii* HAP2 is 1.290 Å (121 pruned atom pairs; across all 425 pairs, the value is 13.539 Å) (Fig. 1a, Supplementary Mov. S1). With *A. thaliana* HAP2, the rmsd value is 1.144 Å (144 pruned atom pairs; across all 428 pairs, the value is 11.134 Å (Fig. 1b, Supplementary Mov. S2). Three structural domains of *B. bovis* HAP2, which, with the exception of DIII, are not co-linear with primary structure of the protein, namely DI, DII & DIII were also identified through structural analysis (Fig. 2).

Schematic representation of the primary sequence of the *B. bovis* HAP2, showing the signal peptide (SP), transmembrane domain (TM), the cytoplasmic domain (Cyt), and the HAP2 structural domains I, II, and III, are represented in Fig. 2. It was predicted that the signal peptide of HAP2 is located between amino acids 1–33, the domains DI, DII, DIII & the stem region (631–682) which constitute its ectodomain (Fig. 2), the transmembrane helix is between 683 and 703.

**Fig. 3:**
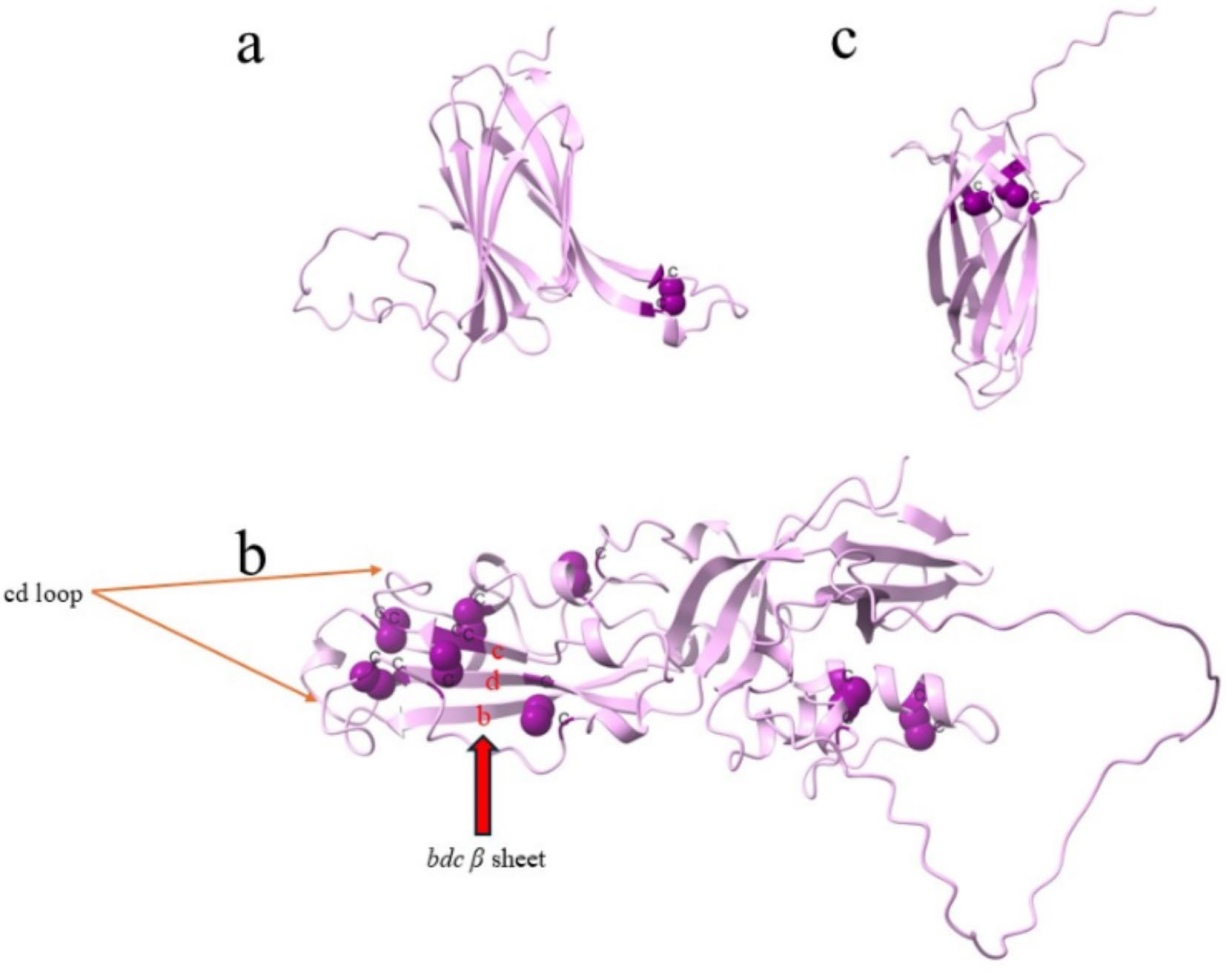
Cysteines & disulfide bonds in *B. bovis* HAP2: (**a**) Domain I, (**b**) Domain II (**c**) Domain III. The purple spheres indicate the disulfide bonds between cysteine residues. The orange arrows in Domain II indicate the *cd* loop and the red arrow indicates *bdc* β sheet.

**Fig. 4:**
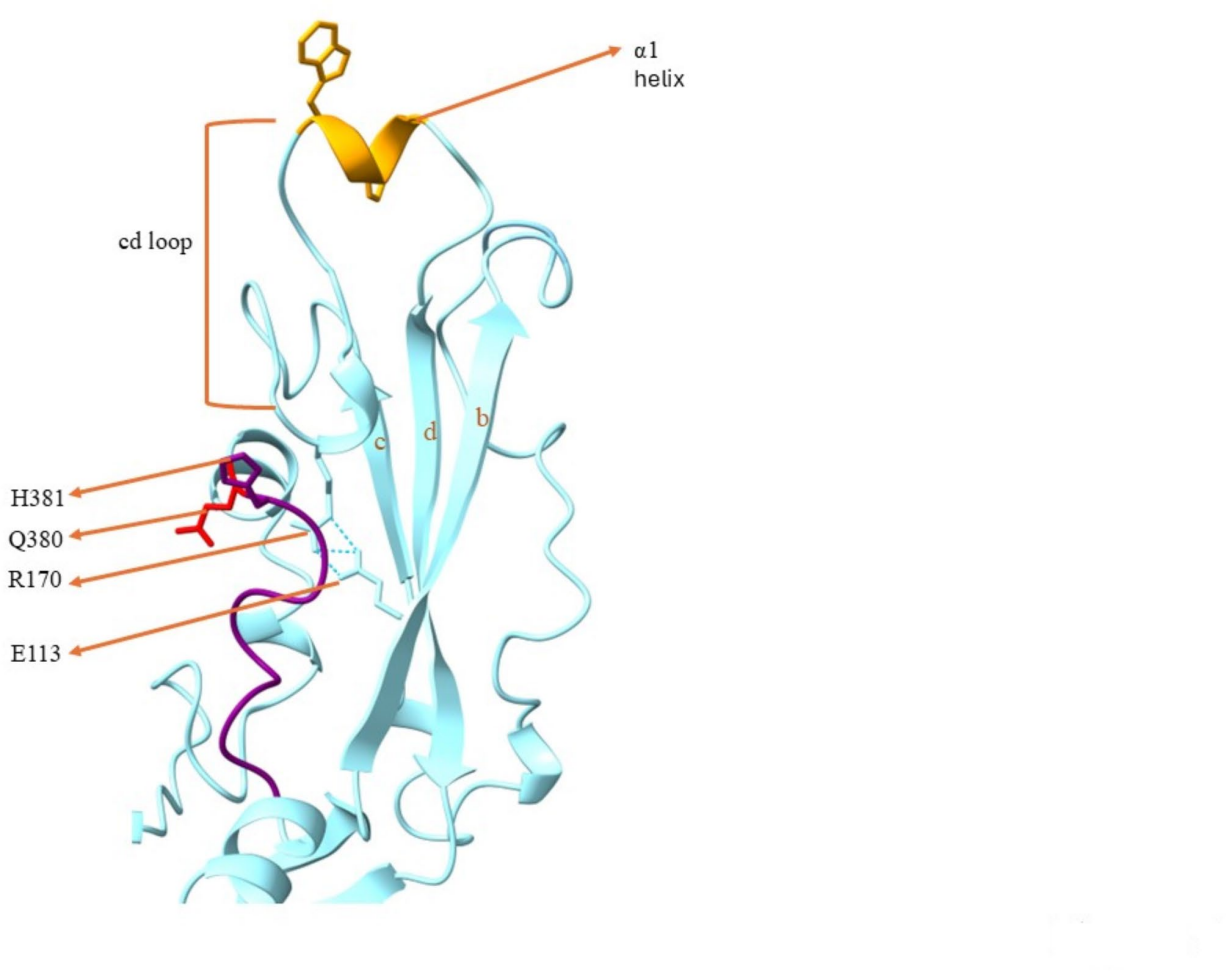
Close-up of *bdc* β sheets of domain II of *B. bovis* HAP2. The α1 helix, the tryptophan and phenylalanine residues are colored orange, the hydrogen bonds are represented as cyan dots, The *ij* loop is in purple color, the conserved histidine residue is colored as purple and the glutamine residue is in red.

Domain I is 160 amino acids(aa) long, has ten beta strands which are anti-parallel and form a β sandwich, and is discontinuous (Figs. 2 and 3a). Domain II has both beta strands and alpha helices comprised of 314 aa and it has two discontinuous parts, flanked by sequences that are a part of domain DI (Figs. 2 and 3b). There are three beta strands at the farthest part of this domain which are labeled as *b*,* d*,*c* (Fig. 4). The loop at the tip of this domain is the predicted fusion loop, called the *cd* loop because it connects c and d β strands (Figs. 3b and 4). At the tip of *cd* connection, one α-helix is located which is named α1 helix in this study. The conserved residues E113 and R170 at the *bdc* sheet region form a salt bridge. The presence of *ij* loop at the *bdc* sheet region is also noticed. In the *ij* loop, Q380 and H381 residues are conserved (Fig. 4). Domain III is smaller compared to DI and DII and is colinear with the primary protein structure. This domain has 124 amino acids spanning from amino acid 507 to 630 of the HAP2 sequence (Figs. 2 and 3c) and is located next to the transmembrane domain of the protein. The beta strands of the Domain III are anti-parallel and form an immunoglobulin-like fold (Fig. 3c)^43,47^.

**Fig. 5:**
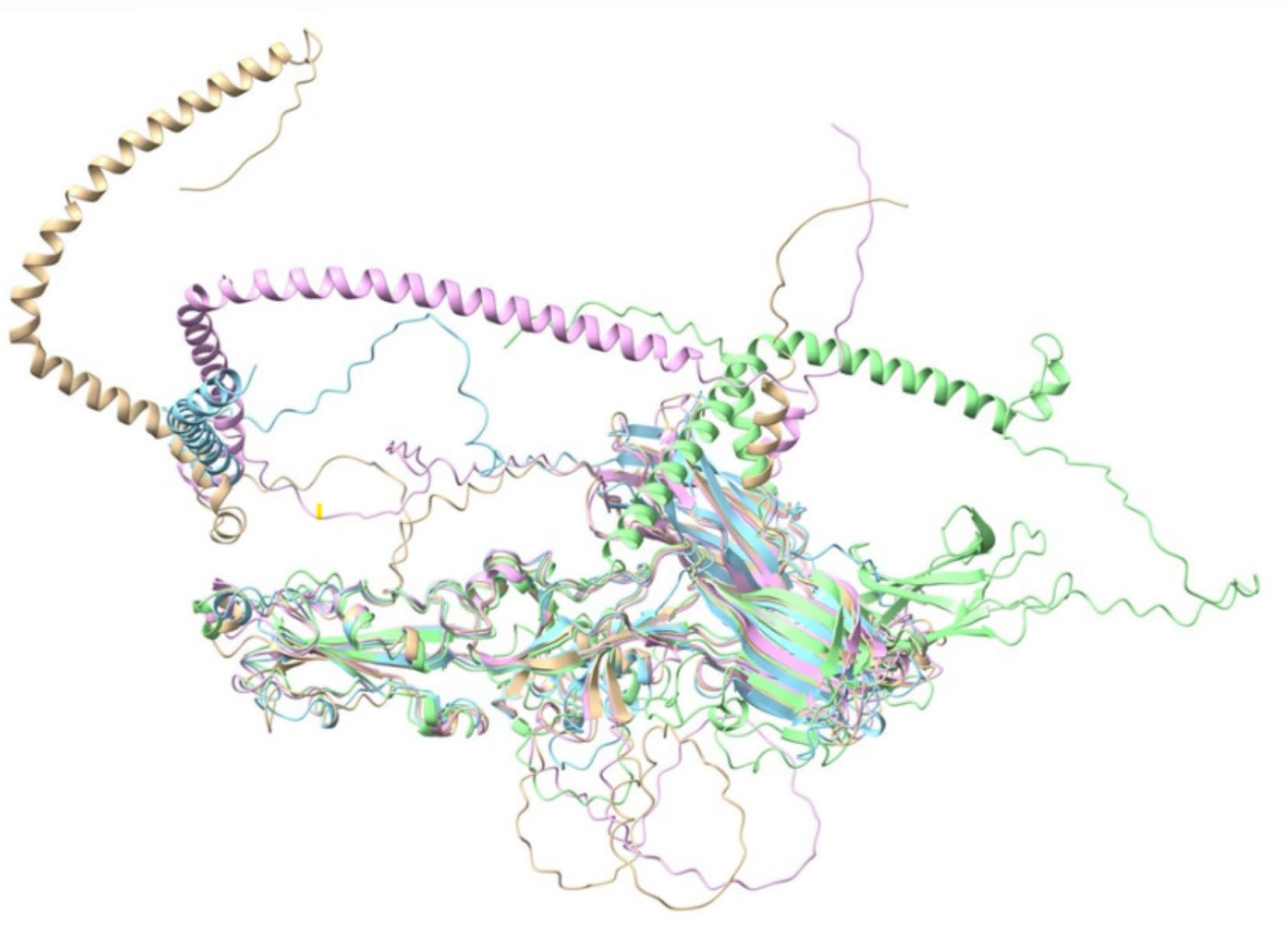
Superimpostion of HAP2 proteins of different *Babesia* parasties. Superimposition of HAP2 of *B. bovis* (pink) with the HAP2 of *B. bigemina* (gold), *B. microti* (cyan) & *B. divergens* (light green).

Structural alignment of *B. bovis* HAP2 was also performed with the predicted HAP2 structures of *B. bigemina*, *B. microti* and *B. divergens*. The rmsd values between *B. bovis* HAP2 and *B. bigemina* HAP2 is 0.922(463 pruned atom pairs; across all 743, the value is 17.782) (Fig. 5 and Supplementary Mov. S3). With *B. microti* HAP2, the rmsd value is 1.051(264 pruned atom pairs; across all 606, the value is 12.109) (Fig. 5 and Supplementary Mov. S3) and with HAP2 homologue of *B. divergens* which is annotated as putative membrane protein, the rmsd value is 1.240(280 pruned atom pairs; across all 695 pairs, it is 48.216) (Fig. 5 and Supplementary Mov. S3). Also, structural superimposition of *B. bovis* HAP2 DIII was done with the crystal structure of HAP2 DIII of *Plasmodium berghei* (Supplementary Fig. S1) and the rmsd value is 0.933(69 pruned atom pairs; across all 108 pairs, the value is 6.827).

### Sequence conservation among HAP2 proteins from different organisms

**Fig. 6:**
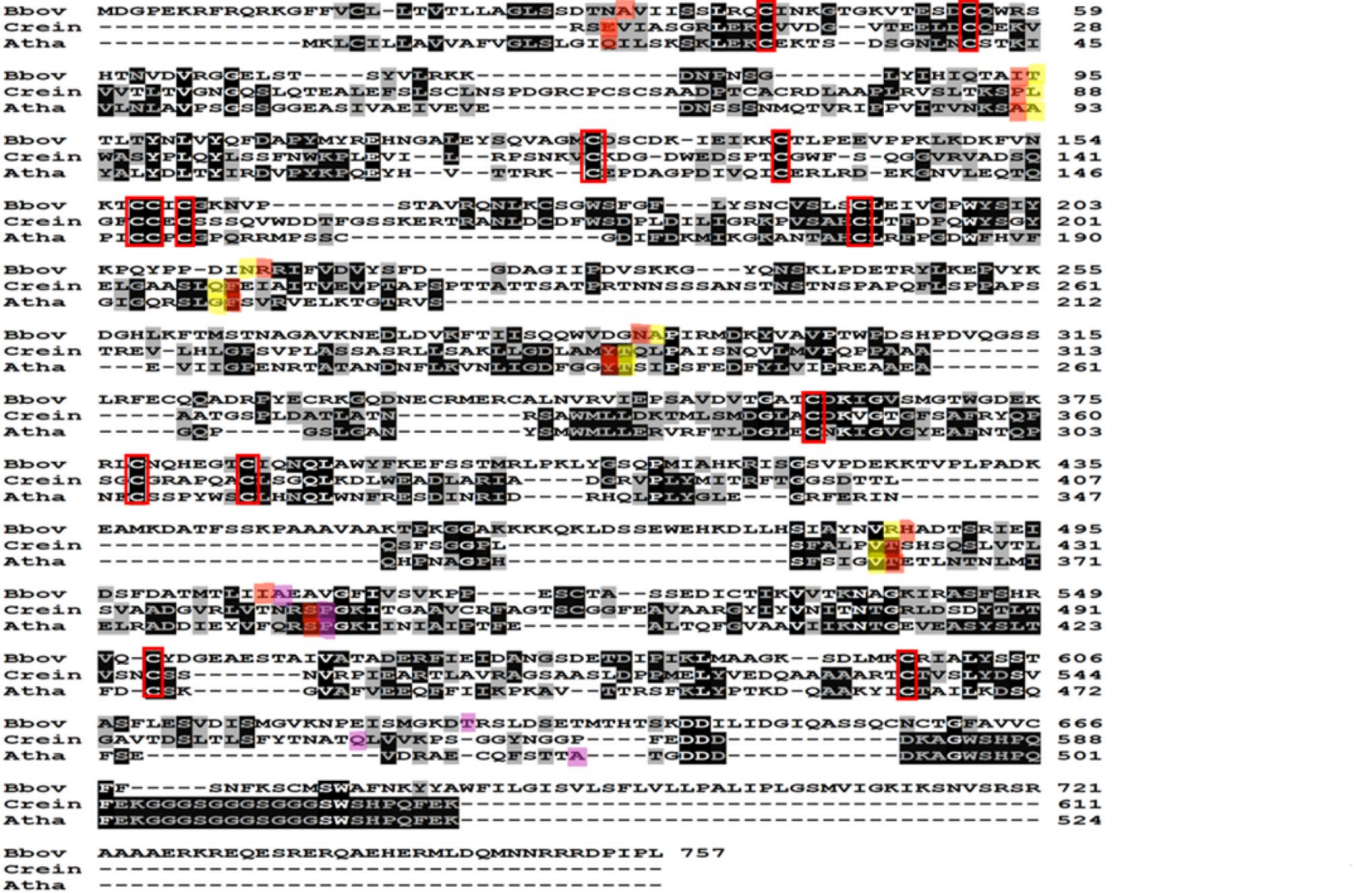
Sequence alignment of HAP2 of *B. bovis* with the HAP2 ectodomains of *C. reinhardtii* & *A. thaliana*. The red highlighted letters indicate the sequences of DI, Yellow highlighted letters indicate DII and Pink highlighted letters indicate DIII. The red rectangular boxes indicate 13 conserved cysteine residues that form disulfide bonds across the three domains.

The sequence alignments of *B. bovis* HAP2, HAP2 ectodomains of *C. reinherdtii* and *A. thaliana* showed overall low sequence similarity (Table 1). The alignment of 13 cysteines among these three sequences was noticeable. However, there are 22 cysteines across the three domains of HAP2 ectodomains of *B. bovis* and *C. reinherdtii*, while only 15 cysteines are present in the *A. thaliana* HAP2 (Fig. 6). Besides, sequence alignments among *B. bovis*, *B. bigemina*,* B. microti* and *B. divergens* indicate that the *B. bovis* HAP2 has 47.7%, 24.3% and 41.6% sequence similarity respectively with its orthologues in these three related parasites. Interestingly, there are 20 cysteines aligned among these four *Babesia spp.* parasites located across their three domains. It is notable that while there are 23 cysteine residues in *B. divergens*,* there* are just 22 cysteines across the domains of *B. bovis*, *B. bigemina* & *B. microti* (Supplementary Fig. S2).

**Table 1:** Percent identity of HAP2 of *B. bovis*(Bbov) with HAP2 ectodomain of *C. reinhardtii* (Crein) & *A. thaliana* (Atha).

Bbov	100%		
Crein	17.5%	100%	33.0%
Atha	18.9%	33.0%	100%
	Bbov	Crein	Atha

### Antibodies from cattle immunized against full size recombinant *B. bovis* HAP2 recognize epitopes in DI, DII, and DIII

**Fig. 7:**
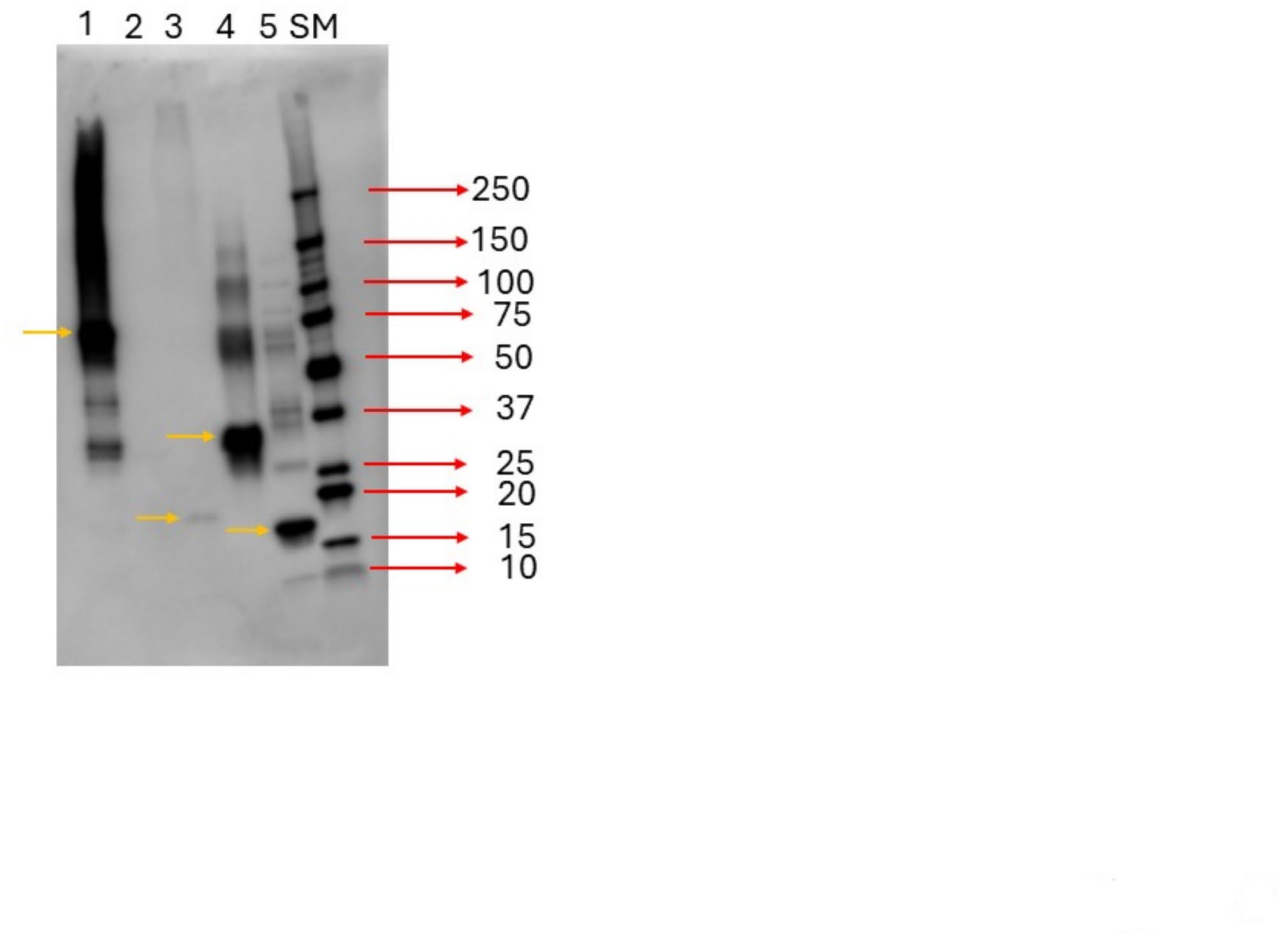
Immunoblot analysis. Immune; Lanes. (1) HAP2; (2) Unrelated control protein; (3) Domain I; (4) Domain II; (5) Domain III. SM = Size Marker. The orange arrows indicate the HAP2 protein and the three domains.

**Fig. 8:**
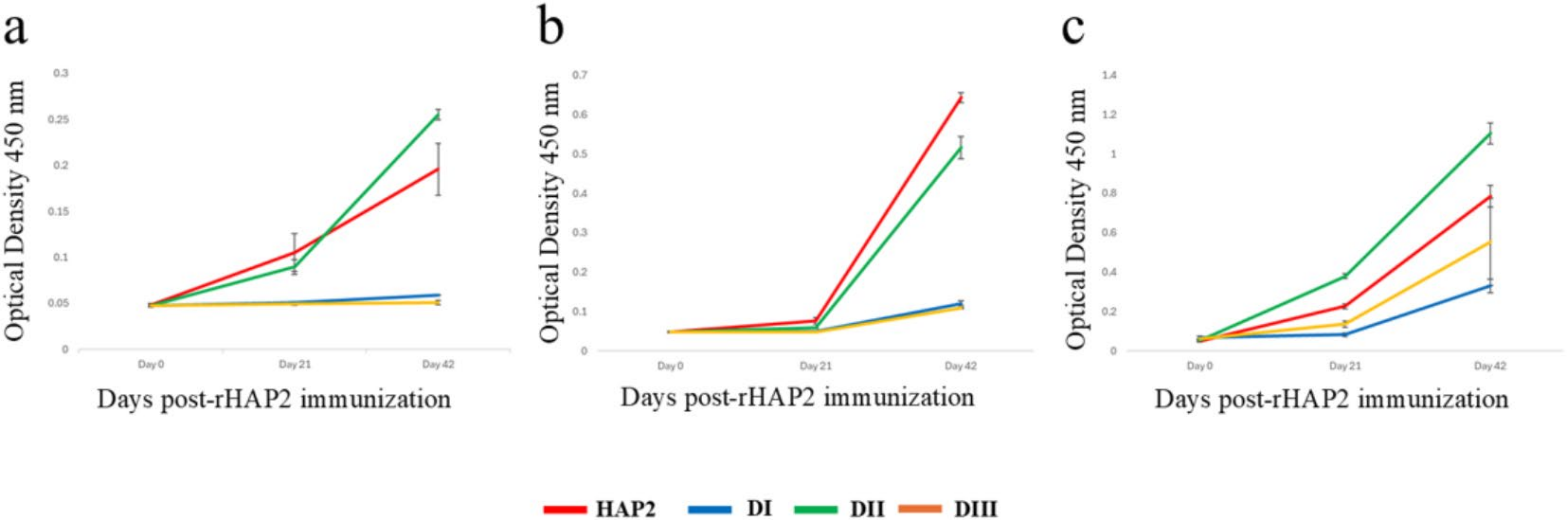
Serological analysis by iELISA using the sera from rHAP2 immunized three animals of Day 0, Day 21 and Day 42 post-immunization. (**a**) Animal 1, (**b**) Animal 2 (**c**) Animal 3. Red, blue, green and orange colored lines represent HAP2, DI, DII and DIII respectively. The error bars represent the standard deviations of the samples at each time point.

In addition to full size recombinant HAP2 *B. bovis*, we expressed and purified three recombinant proteins containing the amino acids sequences representing in full the structural domains DI, DII and DIII. The expected molecular weight of full size HAP2, DI, DII and DIII is ~ 75kD, ~ 19kD, ~ 36kD and ~ 17kD respectively. Immunoblot analysis of recombinant proteins representing the DI, DII and DIII domains using sera from three bovines immunized with full size recombinant HAP2 (rHAP2)^25^ is shown in Fig. 7. As described in Fig. 2, the primary sequences of the DI and DII domains are comprised of discontinuously located amino acids, and therefore their recombinant versions are chimeras that are not strictly and fully colinear with the native HAP2 protein, although they maintain their relative order as they are located in the primary sequence of this protein. The recombinant DI and DII proteins were generated by adding the discontinuous portions together in a synthetic DNA expression construct. Neither rHAP2 nor any of the three recombinant proteins representing the HAP2 structural domains reacted with the pre-immune cattle sera (Supplementary Fig. S3), but rHAP2 and all three domains reacted with the sera from rHAP2 immunized bovine, with differential intensities (Fig. 7, Supplementary Fig. S4). The antibodies in a transmission-protected bovine react strongly with full size HAP2, and domains DII and DIII, but weakly with DI (Lane 3, Fig. 7). Thus, altogether, the results indicate that the three domains contain B-cell epitopes recognized by the bovine immune system and may contribute to the elicitation of transmission blocking immunity by rHAP2. The rHAP2 in this analysis was used as positive control while, one unrelated recombinant protein (*B. bovis* rSA1, Lane 2, Fig. 7) was used as a negative control in these experiments, confirming the specificity of the immunoblots (Fig. 7)^25^. As the domains are Histidine (His)-tagged, the immunoblot analysis was also performed to ensure that the domains were recognized by HRP conjugated His-tagged antibody (Supplementary Fig. S5). In addition, indirect ELISA (iELISA) tests were also performed to examine the response of the domains against the sera from the protected animals (Fig. 8). The iELISA analysis, performed using sera derived from three HAP2 immunized, transmission protected cattle^25^, showed that all three domains were reactive with the sera from three rHAP2-imunized animals, although, again, with different intensities. The antibody response against the domains manifested a sharp increase at 42 dpi (days post immunization of cattle with full size recombinant HAP2) following the second boost immunization with rHAP2. This finding is in accordance with the previous experiment of rHAP2 immunization^25^. Interestingly, and mostly consistent with the immunoblot analysis, the antibody response against the DII domain was significantly higher than against the other two domains (*p* < 0.05) in all three HAP2 immunized and transmission blocking resistant calves tested. No significant differences were detected in the iELISA analysis concerning the levels of antibodies to DI and DIII domains.

## Discussion

*Babesia bovis* is the main agent responsible for bovine babesiosis. Expression of HAP2 is indispensable in the events leading to gamete fusion in this parasite to perpetuate parasite life cycle, and inhibition of HAP2 function through HAP2 immunization of cattle can block the transmission of *B. bovis*^25^, but the mechanisms involved in protection remained unknown. The ability of anti-HAP2 antibodies to prevent zygote formation of *B. bigemina* was also shown^49^. The elucidation of the *B. bovis* HAP2 structure is important for understanding HAP2 function, the transmission blocking activity elicited by HAP2 immunization of cattle, and mechanisms involved in sexual reproduction of the parasite. There are structural studies on the HAP2/fusion protein of different microorganisms and species^38,41–47^, but to our knowledge, so far, there is no structural study performed on the *B. bovis* HAP2. Here in this study, we performed structural analysis of HAP2 using various bioinformatics approaches, and compared the antigenicity of each of the domains, using two distinct experimental approaches.

The structural analysis showed that the rmsd value is less than 2 angstrom of pruned atom pairs that indicates there is significant structural similarity of *B. bovis* HAP2 with that of *C. reinhardtii* HAP2 ectodomain and *A. thaliana* HAP2 ectodomain. In addition, this study showed that there are three domains in the *B. bovis* HAP2 and those domains are predicted to be extracellular. Therefore, the results emerging from this investigation are in full agreement with the previous structural studies performed on HAP2/fusion proteins that showed that HAP2/fusion protein has three extracellular domains^41–47^. This structural similarity was also found among the HAP2 of *B. bovis*, *B. microti*, *B. bigemina* and *B. divergens*, therefore, the HAP2 proteins in these related parasites also have three extracellular domains. However, low sequence similarity of HAP2 orthologues of *C. reinhardtii*, *A. thaliana* and *B. bovis* was noticed, but this is not surprising since these are very distantly related organisms. Nevertheless, 13 cysteine residues were aligned across the three domains of the HAP2 protein among these three organisms, suggesting structural conservation requirements for conserved HAP2 functions. Consistent with their phylogenetic relationships^50^, it was observed from the sequence alignment of HAP2 among four *Babesia* parasites that *B. bovis* HAP2 has relatively high similarity with the *B. bigemina* and *B. divergens* HAPs, but less similarity with the *B. microti* HAP2. However, all these four organisms can undergo sexual reproduction in the midgut of their tick vectors, regardless of their *sensu stricto* or *sensu lato* classification.

Sequence conservation is important to define conserved amino acid sequences that are essential for HAP2 functions. Data from sequence comparisons of HAP2 of four *Babesia* parasites revealed that 20 cysteine residues located across the three domains of the ectodomain regions of these parasites were aligned. Cysteine residues form disulfide bonds and help to maintain structural stability^51^. The highly significant structural similarity found among these HAP2 might be due, at least in part, to the strict conservation of these cysteine residues.

Domain I of *B. bovis* HAP2 has ten anti-parallel β strands and there is only one disulfide bond found that is located between the two longest β strands. This disulfide bond is made of Cysteine (Cys)-42 and Cys-55 residues. In *A. thaliana* HAP2 DI, one disulfide bond is shown in the same location^44^. Though, in *C. reinhardtii* HAP2 DI, there is one disulfide bond in a similar region, there are other three disulfide bonds in the loop connecting these two beta strands that form ladder like conformation^46^. DI and DIII of *B. bovis* are connected by a linker. Domain II of *B. bovis* HAP2 is the longest domain of this protein. The *cd* loop is located at the tip of domain II. The most disulfide bonds of *B. bovis* HAP2 are found in the DII domain. There are 8 disulfide bonds in the DII domain, of which 6 are in the well conserved *bdc* sheet region, highlighting an important functional role for this region. Whereas, in *C. reinhardtii* and *A. thaliana* HAP2 DII, there are 5 disulfide bonds which are in the *bdc* sheet region^43,46,47^. The α1 helix at the tip of the *cd* loop is amphipathic. Also, in *A. thaliana*, there is one amphipathic α-helix in the fusion loop. But, while in *C. reinhardtii*, there are two fusion loops with two amphipathic α-helices, in *Trypanosome cruzi*, there is an absence of this helix, and it has three short fusion loops^44^. Interestingly, the α1 helix is much shorter than the helix of *A. thaliana* and the fusion helices of *C. reinhardtii*. The α1 helix of *B. bovis* HAP2 contains Phenylalanine(F) and Tryptophan(W) which may form a non-polar surface. This non-polar surface in *B. bovis* HAP2 is likely essential for insertion into the target membrane, leading to gamete fusion. The presence of a non-polar surface at the fusion helices was also noticed in *C. reinhardtii* and *A. thaliana*. The loop of *bc* strand connection is located a little bit lower relative to the alpha helix, indicating that only the *cd* loop may participate in the membrane insertion of this HAP2, without involving the *bc* loop. This structural arrangement is similar to *A. thaliana*, where the α1 of *cd* loop participates in the membrane insertion. In *C. reinhardtii* HAP2, two alpha helices at *cd* loop take part in membrane insertion^43–46^. The salt bridge formed between the conserved arginine and glutamic acid is strictly conserved among HAP2/fusion proteins. The salt bridge connects different regions which are responsible for membrane insertion around the *bdc* sheet. The salt bridge is formed between R185-E126 in *C. reinhardtii* HAP2, between R163-E117 in *A. thaliana* HAP2. The *ij* loop which is also conserved among HAP2/fusion proteins is observed in the *B. bovis* HAP2 as well, as shown in Fig. 4. The Q380 residue is the conserved glutamine residue at the *ij* loop of *B. bovis* HAP2 whereas, the conserved glutamine is Q379 residue in *C. reinhardtii* HAP2 and in *A. thaliana* HAP2, it is Q308 residue (Fig. 4)^43,44,46^. There is a histidine present in this *ij* loop (Fig. 4). The presence of this histidine residue was also reported in the E1 fusion protein of flavivirus and alphavirus. It was shown that the mutation of histidine to alanine makes the virus non-infectious and membrane fusion was completely abrogated, and so, it may also be functionally critical in *Babesia* parasites. The histidine residue at the *ij* loop is present in the *A.* thaliana HAP2 but, not in *C. reinhardtii*^40,43,44,46,52^.

The shortest domain of the *B. bovis* HAP2 is domain III. The seven β strands of this domain are arranged in two β sheets. This domain has two disulfide bonds, one bond is made of Cys-522 and Cys-530, while another is made of Cys-552 and Cys-598 residues. In the case of *C. reinhardtii* HAP2 DIII, there are also two disulfide bonds present. However, and surprisingly, only one disulfide bond and one free cysteine is present in the *A. thaliana* HAP2 DIII. The long disulfide bond comprised of Cys-552 and Cys-598 residues in *B. bovis* is conserved, as it is also present in *C. reinhardtii*, *A. thaliana* and in *P. berghei*^43–46^. Importantly, both predicted disulfide bonds of *B. bovis* HAP2 DIII are conserved with the disulfide bonds experimentally determined for the *P. berghei* DIII domain (Supplementary Fig. S1). It was shown in various studies that the folding back movement of domain III, together with the stem region, is a critical step during the fusion^38–40^. The presence of this domain and stem region might also play a similar pivotal role in the gamete fusion of *B. bovis*.

The immunoblot analysis using rHAP2 immunized cattle sera showed that DI, DII, and DIII are recognized by antibodies from the immune bovine sera, so they all contain B-cell epitopes recognized by the bovine immune system. The iELISA revealed that DII domain has robust antibody response compared to the other two domains, while the domain DI has the lowest response when compared in immunoblots. However DI and DIII appear to have similar levels of reactivity in the iELISA tests, but these data cannot be compared with the immunoblots, since the iELISA was performed using sera from three distinct immunized animals, whereas the immunoblot only used a single animal. The antibody response of domain DII at 42 dpi was significantly higher than the antibody responses detected against the DI and DIII domains for all three vaccinated animals. Based on this analysis, and regardless of the differences in the tests used, it is noted that the domain DII might have higher potential in the elicitation of transmission bocking immunity, and this should be further demonstrated experimentally.

It was shown that antibodies targeting different domains/regions of HAP2 were able to block gamete/membrane fusion at different magnitudes. For instance, targeting *cd* loop of HAP2 of *P. berghei* and *P. falciparum*^35,37^, domain III of HAP2 of *P. berghei*^47^, residues 231–459 of *P. vivax*^36^, mutating the non-polar residues of fusion helix of *A. thaliana* HAP2, mutating the key arginine residue of salt bridge of *A. thaliana* HAP2^44^, multiple regions of DIII domain of E1 fusion protein of Zika virus^42^, mutating histidine residue of E1 protein of Semliki Forest virus^52^, Epitope of domain III of Dengue virus E1 protein^41,48^, among others. Based on previous findings on domain II and the fusion loop, along with our comparative structural analysis presented hereby, it is possible to speculate that domain II and the fusion loop could play a pivotal functional role in trimerization and thereby, gamete fusion. These data suggest the need of future experiments to confirm whether these regions could be essential for *Babesia* to complete its life cycle within the tick vectors. Furthermore, we suggest that the functional role of these conserved residues could be further verified by using current gene editing methods^53,54^. In addition, this domain could be an effective target to avoid *Babesia* gametes fusion and, consequently, prevent tick infection. In addition, the data emerging from this study would help in defining the HAP2 of other tick-borne parasites that utilize the same mechanisms infect arthropods to perpetuate their life cycle.

In conclusion, similar to other organisms, HAP2 of *B. bovis* contains three domains, DI, DII and DIII, adopting a conserved structure. Recombinant versions of the three domains are differentially recognized by antibodies in sera from cattle immunized with full size recombinant HAP2, demonstrating that they all contain B-cell epitopes. This study will facilitate further investigations of the HAP2 proteins of other *Babesia* parasites responsible for bovine babesiosis and focus on determining what HAP2 domains are able to elicit transmission blocking immunity when used in cattle immunization trials.

## Materials and methods

### Bioinformatic analysis

The predicted 3D structures of HAP2 of *B. bovis* (AF-A7ANV4), *B. bigemina*(AF-A0A061DA61)& *B. microti* (AF-A0A1R4ABY1) were derived from AlphaFold(AF) database. The 3D structure of HAP2 of *B. divergens*(KAK1933072.1) was not available at the AF database, so it was modelled using AlphaFold2 via Google Colaboratory^55–57^. The HAP2 structures of *C. reinhardtii* (PDB ID: 5MF1),*A. thaliana* (PDB ID: 5OW3) and HAP2 DIII of *P. berghei* (PDB ID:7LR3) were obtained from Protein Data Bank (PDB). The structure alignment, analysis, rendering & visualization were performed using ChimeraX^58^. The sequence alignment was done using Clustal Omega^59^. The signal peptide and transmembrane domains were predicted using the DeepTMHMM tool (https://dtu.biolib.com/DeepTMHMM).

### Recombinant protein and domains production

The production and purification of recombinant *B. bovis* HAP2 protein were described previously^25^. The purity was 95%. The sequences of the domains I, II & III were determined from structural analysis. The sequences of the domains I and II are discontinuous. The amino acid sequences from 33 to 94, 213–290, 487–506 were used to generate recombinant domain I, whereas the amino acid sequences from 95 to 212, 291–486 were used to generate recombinant domain II. The recombinant domain III was generated using the 507–630 amino acid sequences. The recombinant domains I, II & III were produced and purified commercially. All the domains were expressed with 6His tagged into *E. coli* BL21 Star™ (DE3) competent cells. The codon optimized sequence of each domain was cloned into pET(30a+) vector (Supplementary Fig. S6). The domains were purified form the inclusion bodies followed by the expression through using Ni column with the purity of $\:\ge\:$70%, $\:\ge\:$90%, $\:\ge\:$80% respectively (GenScript, Piscataway, NJ, USA). Expected molecular weights of the domains were determined by SDS-PAGE (Supplementary Fig. S7).

### Immunoblot analysis

Firstly, the HAP2 protein and domains (1.25 µg/lane) were loaded and ran through 4–20% Mini-PROTEAN^®^ TGX™ Precast Gels (Bio-Rad Laboratories, Hercules, CA), using 5X Sample Buffer containing 10% 2-Mercaptoethanol (GenScript, Piscataway, NJ, USA). Then, the proteins were transferred to the nitrocellulose membranes using iBlot 2™ (Invitrogen, Waltham, MA). The membranes were blocked overnight in 5% milk 4⁰C. After overnight blocking, the membranes were incubated for 1 h in rocking with either pre-immune or HAP2 immunized bovine sera with the 1:10 dilution in 5% milk solution at room temperature. After incubation, the membranes were washed three times with 1xPBS with 0.1% Tween 20 (PBS-T). Then, the membranes were incubated with HRP-conjugated anti-bovine IgG secondary antibody with a 1:5000 dilution in 5% milk for one hour rocking at room temperature. After that, the membranes were washed three times with PBS-T, then, the detection substrates (Prometheus Protein Biology Products 20-300B ProSignal^®^ Pico ECL Reagent by Genesee Scientific, El Cajon, CA, USA) were sprayed onto the membranes and imaging was performed using Azure™ Imaging System (Azure Biosystems, Dublin, CA). The immunoblot in Supplementary Figure S5 was done using 1:7000 dilution of HRP-conjugated His-tagged mouse monoclonal antibody (Proteintech, Rosemont, IL, USA).

### ELISA analysis

Indirect ELISA (iELISA) was performed of these three domains and HAP2 protein. 96-well Immulon™ 2HB microtiter plate (Thermo Fisher Scientific, Waltham, MA) was coated overnight with 50 µl per well of diluted HAP2, DI, DII and DIII domains(0.05µM/ml) at 4⁰C. Then, the plate was blocked with 200 µl of Blocker™ Casein in PBS (Thermo Fisher Scientific, Waltham, MA) for one hour at room temperature. The *E. coli* lysate was added with the blocking solution to suppress the binding of unwanted proteins. After blocking, the bovine serum was diluted in 1:1000 in 1x PBST (SeraCare, Milford, MA) and added 50 µl per well. The plate was incubated for one hour at room temperature. After that, the plate was washed three times with 1x PBST. Then, the plate was incubated with HRP-conjugated anti-bovine IgG secondary antibody with a 1:10000 dilution for 1 h at room temperature. The plate was then washed three times with the same washing buffer. Afterward, 100 µl of SureBlue™ TMB (SeraCare, Milford, MA) was added to each well and incubated for 5 min in the dark. Then, 100 µl of TMB Stop Solution (SeraCare, Milford, MA) was added to each well. The absorbance was measured at 450 nm using the SpectraMax^®^ 190 plate reader (Molecular Devices, San Jose, CA). The statistical analysis was performed by Student’s t-test.

## Electronic supplementary material

Below is the link to the electronic supplementary material.

Supplementary Material 1

## Data Availability

All data is provided within the manuscript or supplementary information files.

## Abbreviations

TBV: transmission blocking vaccines
HAP2: Hapless 2
GCS 1: Generative cell specific 1
