# Supplementary material for: Structural and antigenic characterization of Babesia Bovis HAP2 domains
Source: Sci Rep. 2025 Mar 5;15:7781. doi: 10.1038/s41598-025-91359-4 (PMC11882828; doi:10.1038/s41598-025-91359-4)
Supplement: Supplementary file 1 — Supplementary Material 1 [file 41598_2025_91359_MOESM1_ESM.pptx]

## Slide 1
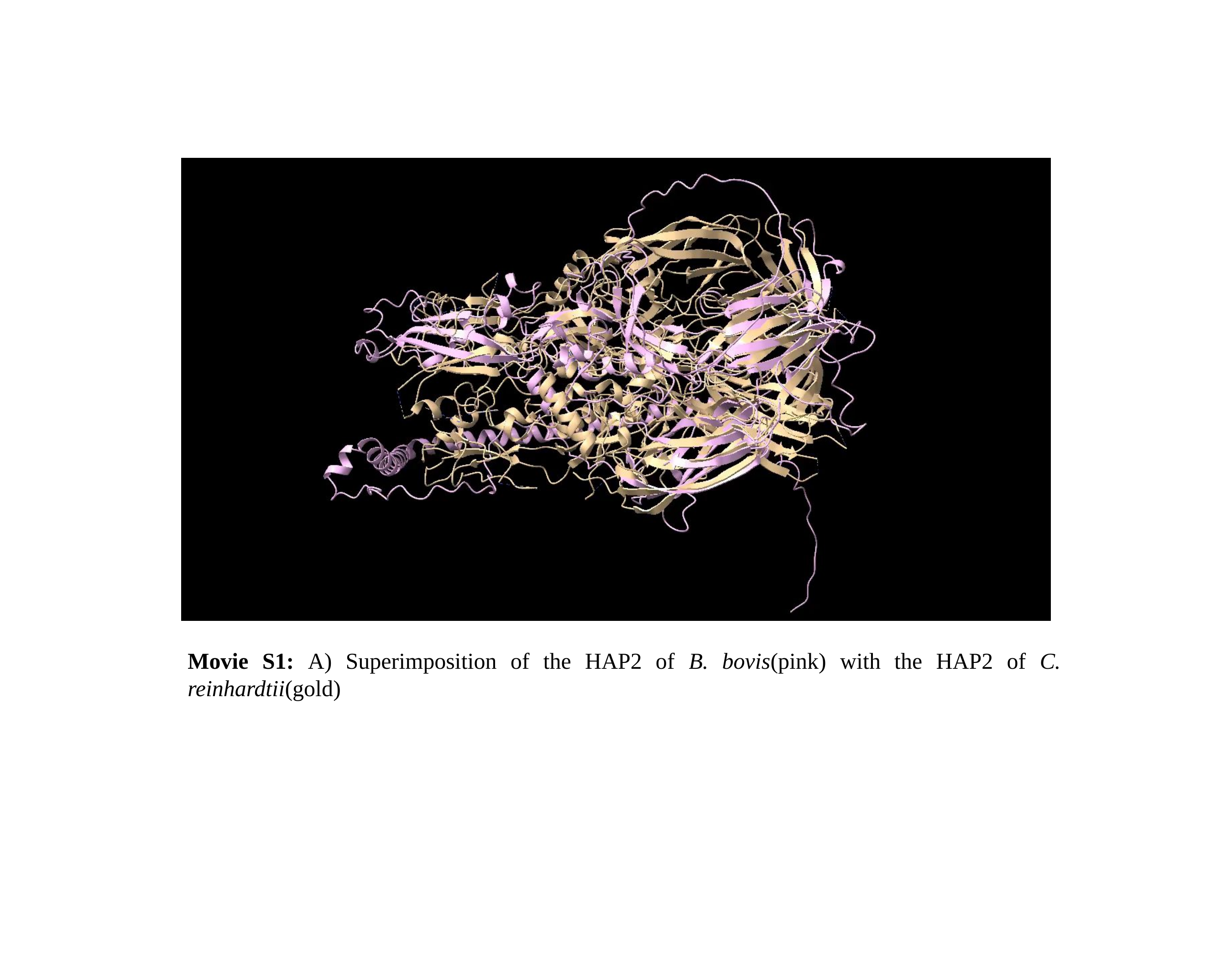

Movie S1: A) Superimposition of the HAP2 of B. bovis(pink) with the HAP2 of C. reinhardtii(gold)

## Slide 2
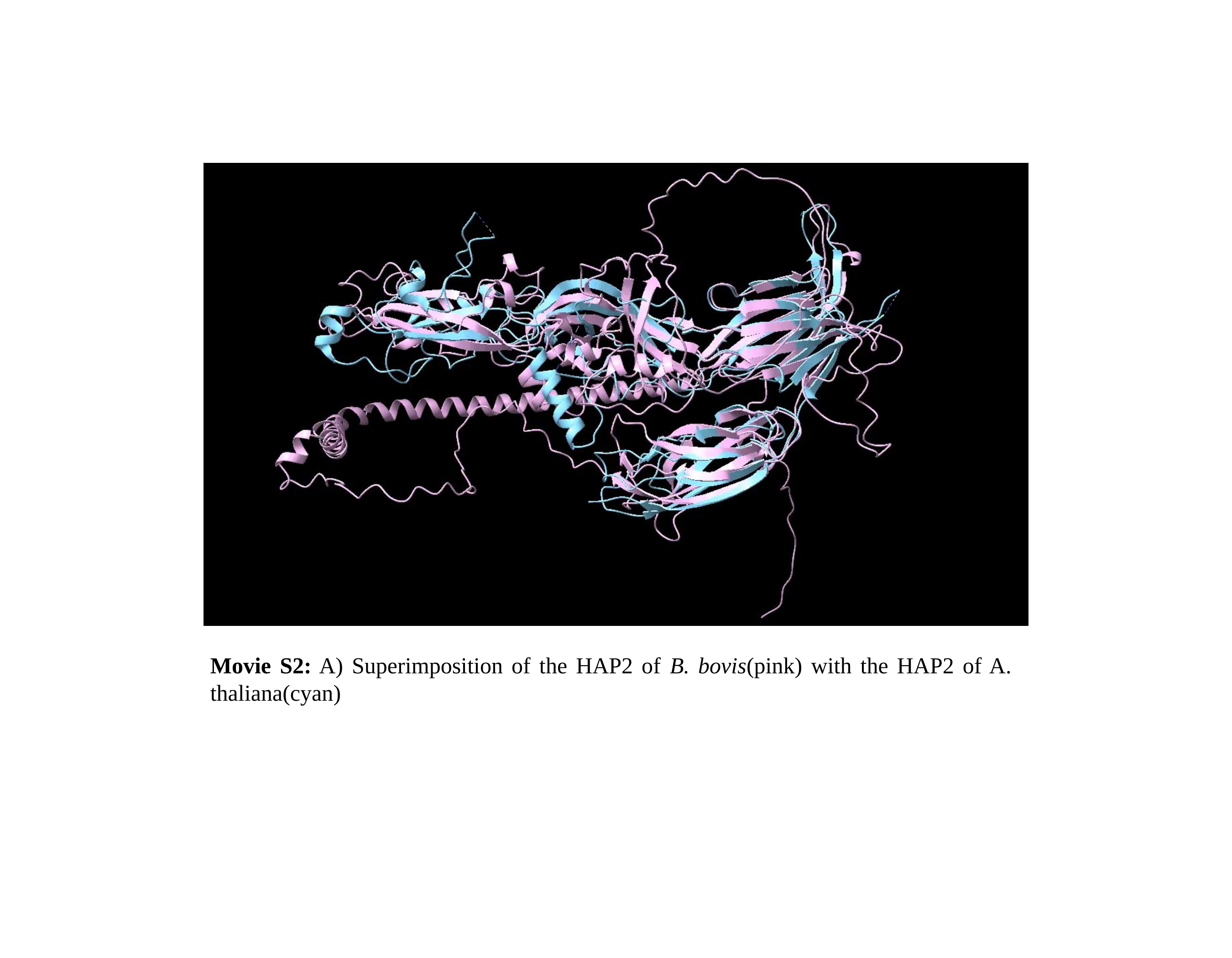

Movie S2: A) Superimposition of the HAP2 of B. bovis(pink) with the HAP2 of A. thaliana(cyan)

## Slide 3
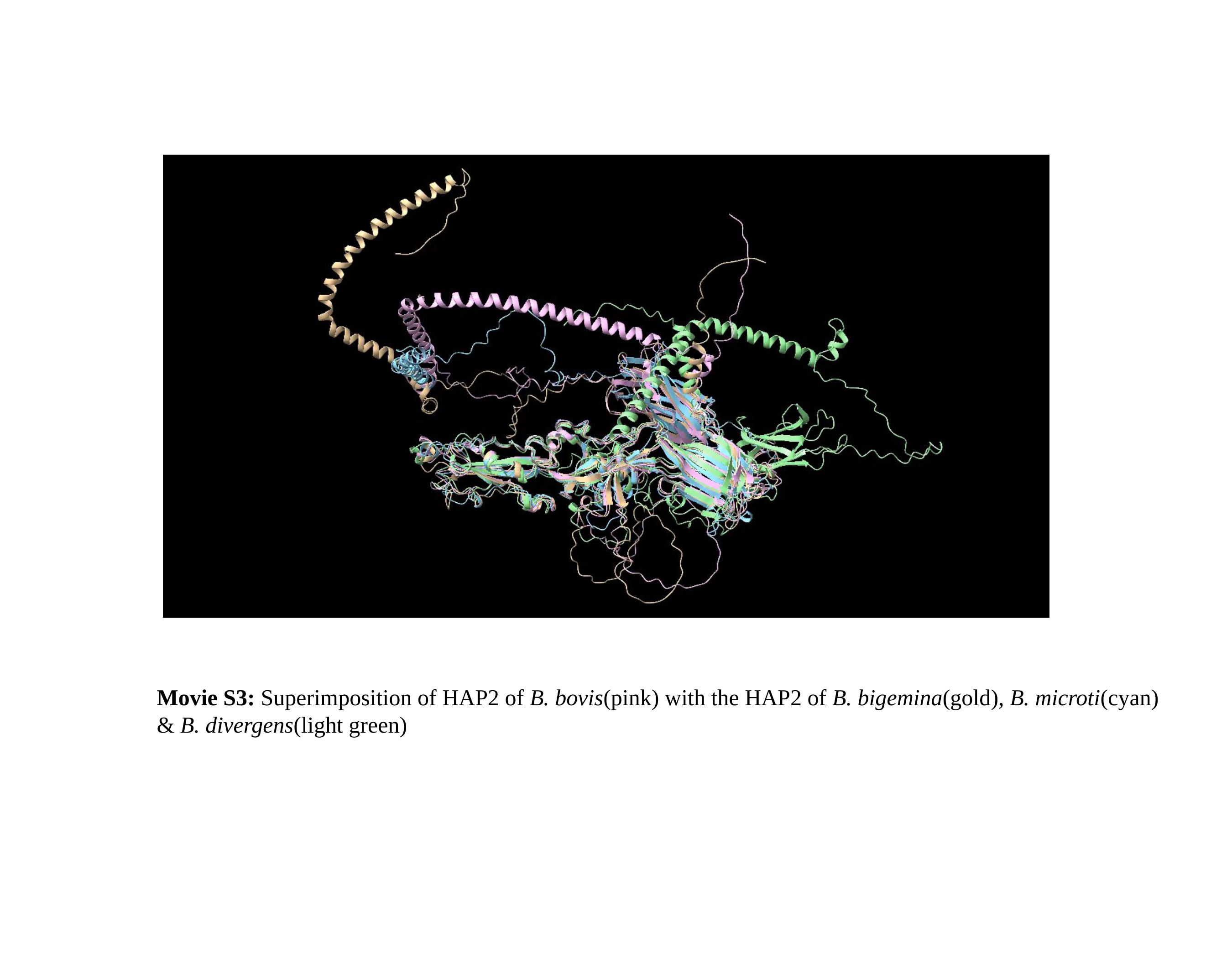

Movie S3: Superimposition of HAP2 of B. bovis(pink) with the HAP2 of B. bigemina(gold), B. microti(cyan) & B. divergens(light green)

## Slide 4
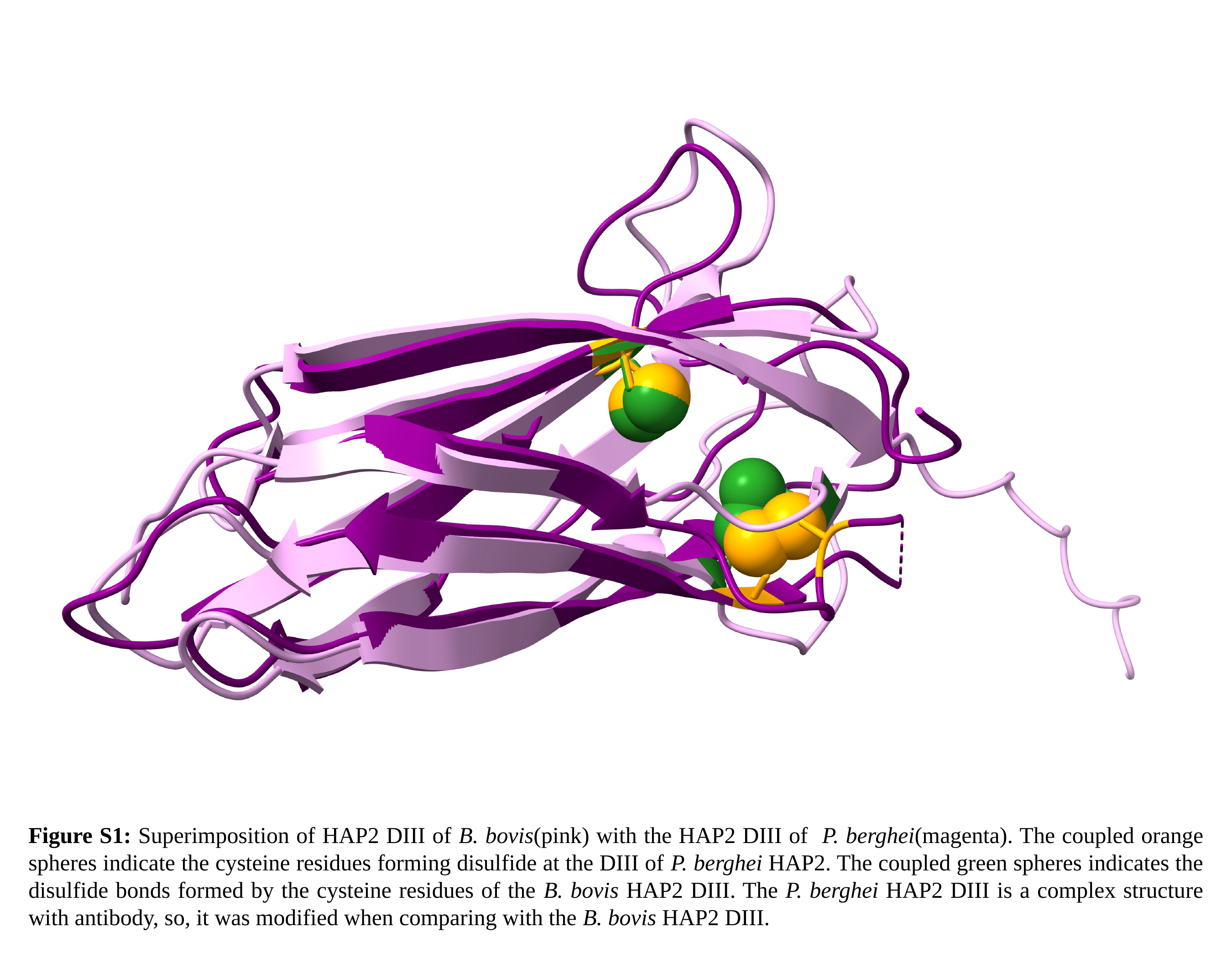

Figure S1: Superimposition of HAP2 DIII of B. bovis(pink) with the HAP2 DIII of P. berghei(magenta). The coupled orange spheres indicate the cysteine residues forming disulfide at the DIII of P. berghei HAP2. The coupled green spheres indicates the disulfide bonds formed by the cysteine residues of the B. bovis HAP2 DIII. The P. berghei HAP2 DIII is a complex structure with antibody, so, it was modified when comparing with the B. bovis HAP2 DIII.

## Slide 5
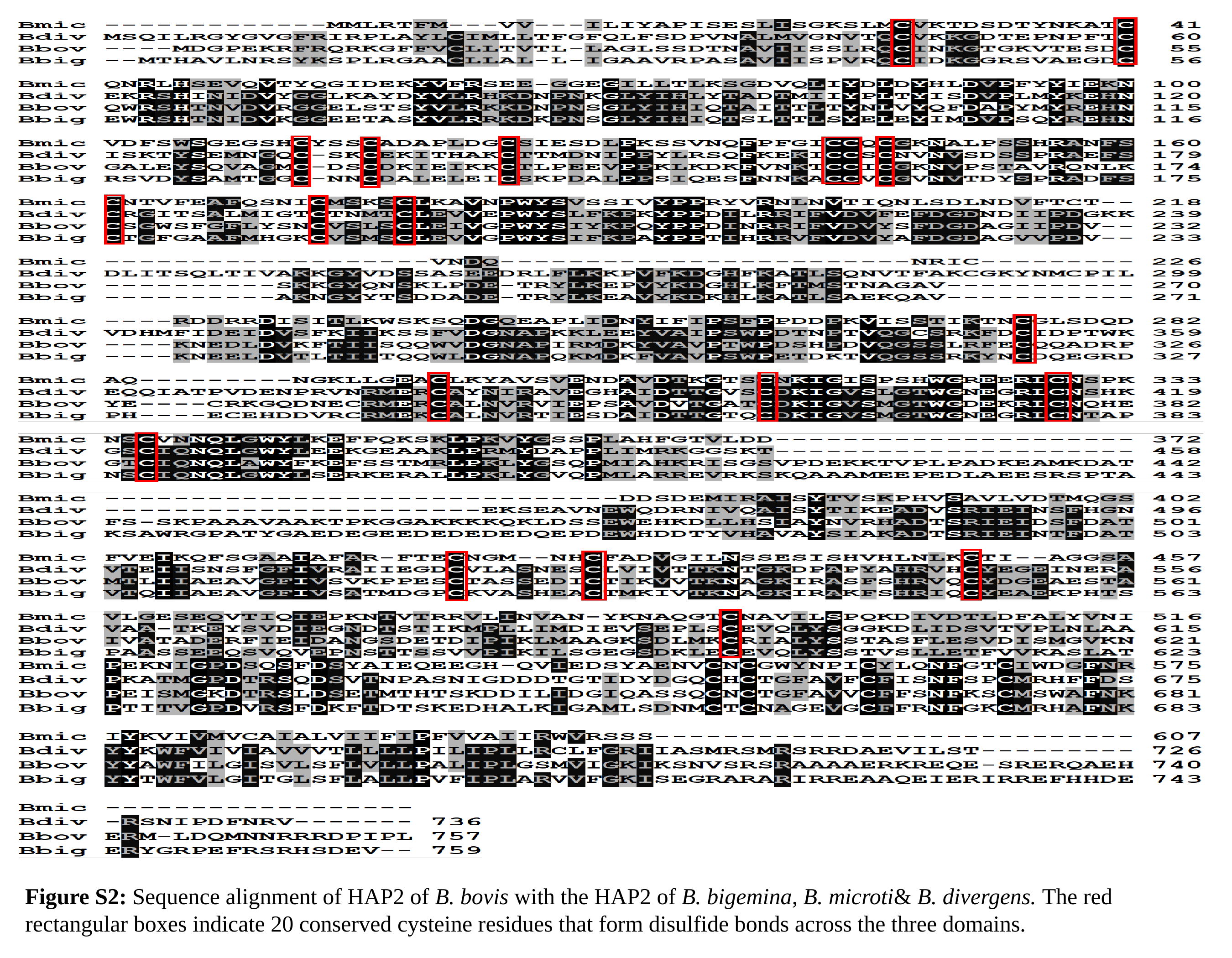

Figure S2: Sequence alignment of HAP2 of B. bovis with the HAP2 of B. bigemina, B. microti& B. divergens. The red rectangular boxes indicate 20 conserved cysteine residues that form disulfide bonds across the three domains.

## Slide 6
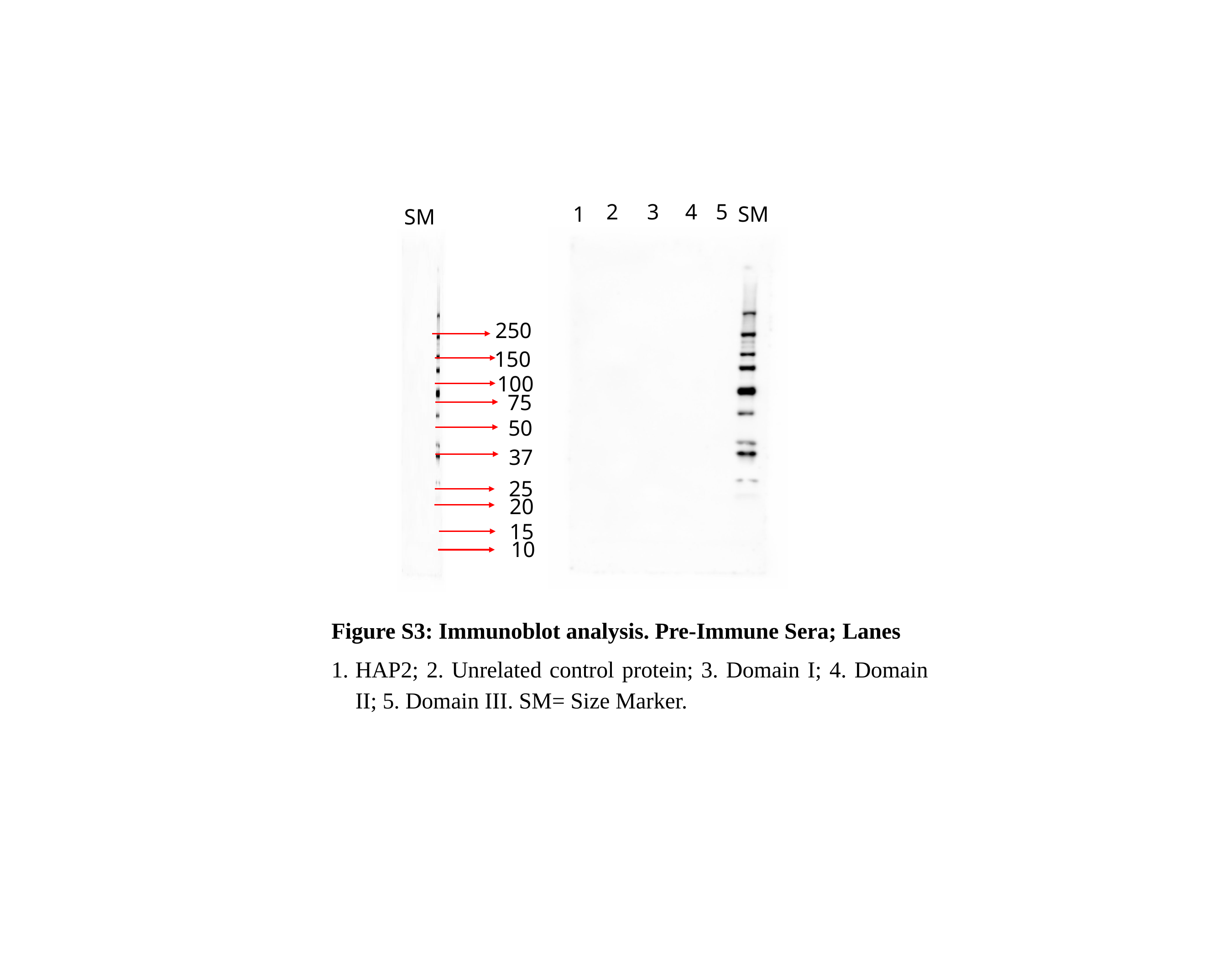

2
3
4
5
SM
1
SM
250
150
100
75
50
37
25
20
15
10
Figure S3: Immunoblot analysis. Pre-Immune Sera; Lanes
HAP2; 2. Unrelated control protein; 3. Domain I; 4. Domain II; 5. Domain III. SM= Size Marker.

## Slide 7
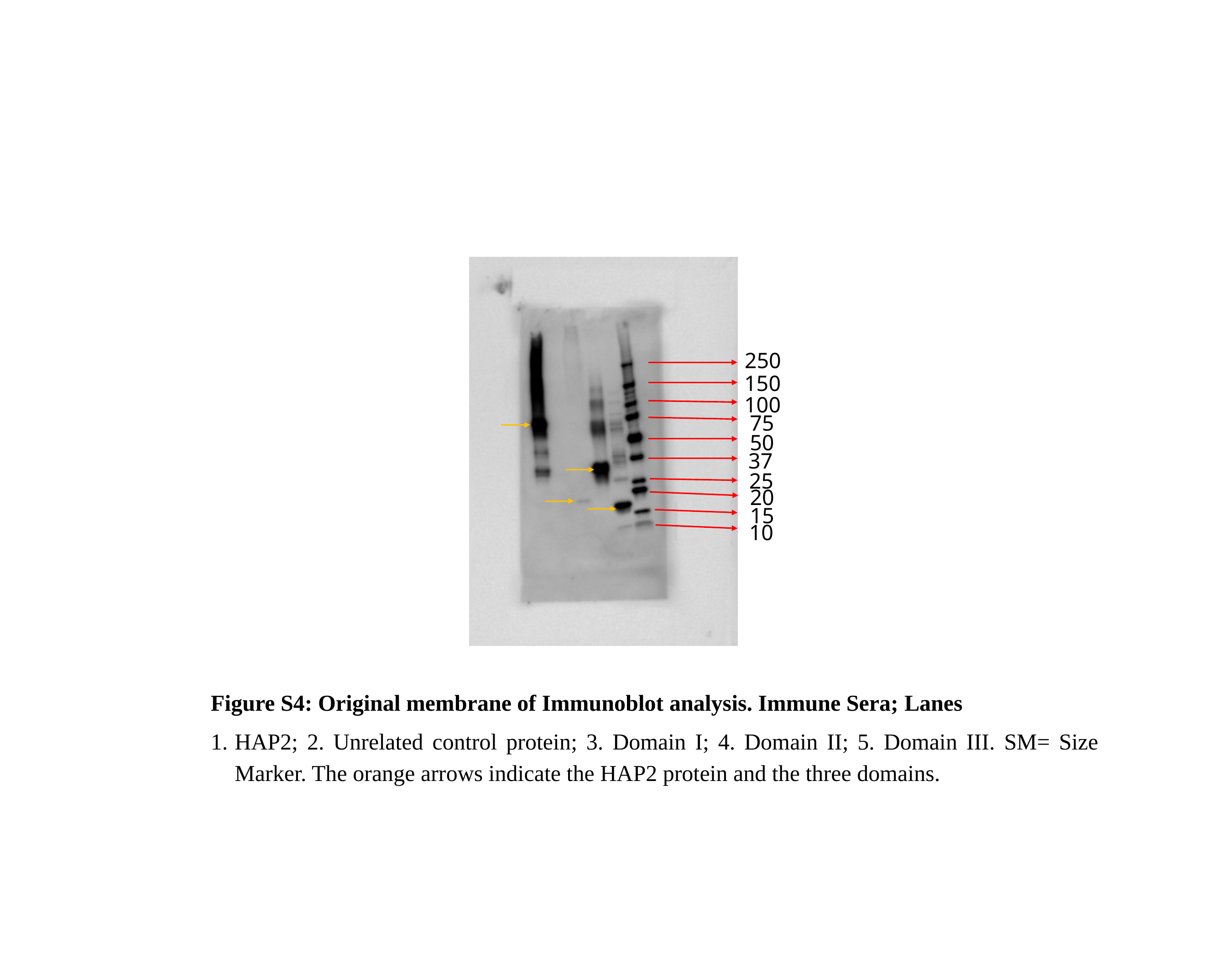

250
150
100
75
50
37
25
20
15
10
Figure S4: Original membrane of Immunoblot analysis. Immune Sera; Lanes
HAP2; 2. Unrelated control protein; 3. Domain I; 4. Domain II; 5. Domain III. SM= Size Marker. The orange arrows indicate the HAP2 protein and the three domains.

## Slide 8
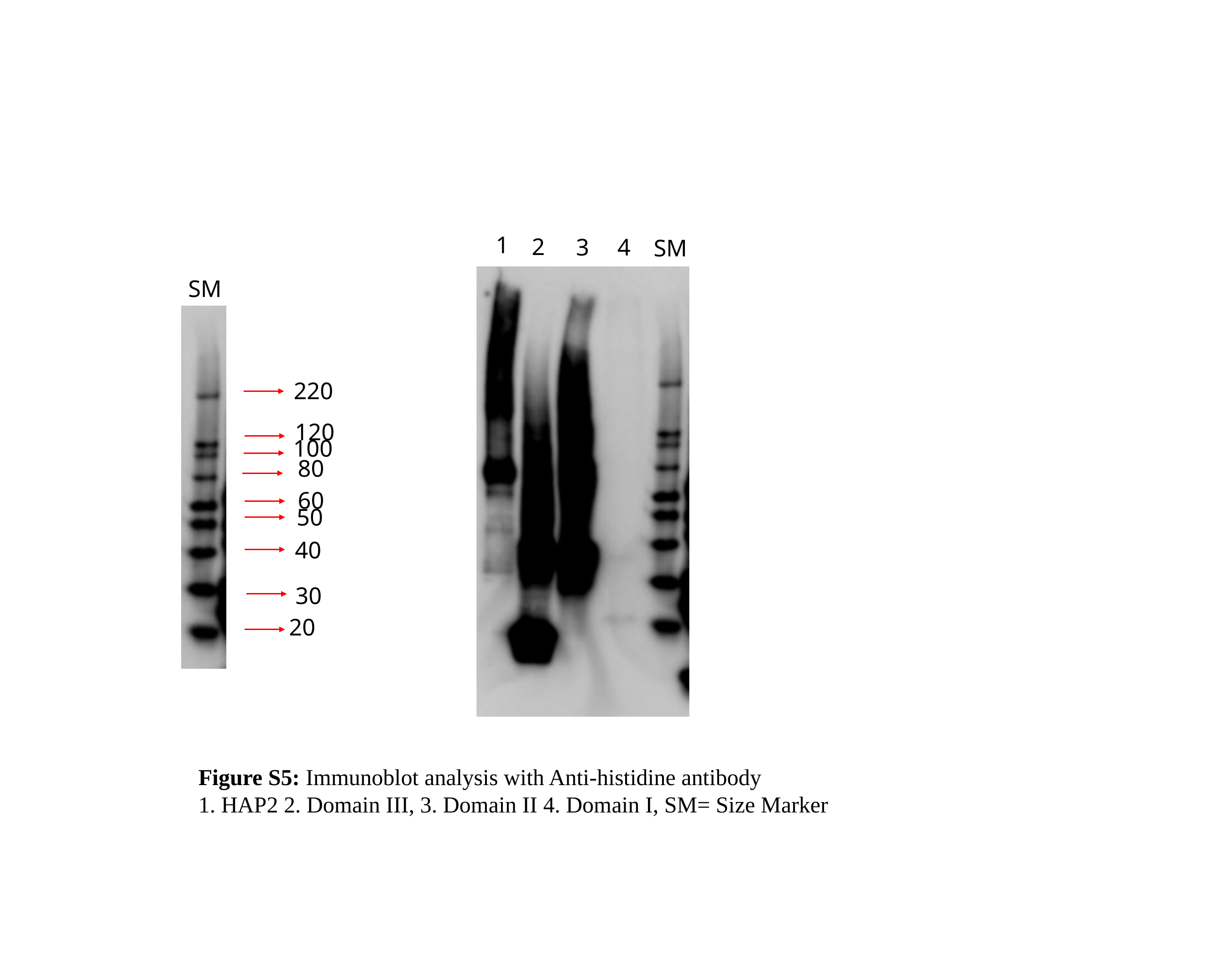

1
2
3
4
SM
SM
220
120
100
80
60
50
40
30
20
Figure S5: Immunoblot analysis with Anti-histidine antibody
1. HAP2 2. Domain III, 3. Domain II 4. Domain I, SM= Size Marker

## Slide 9
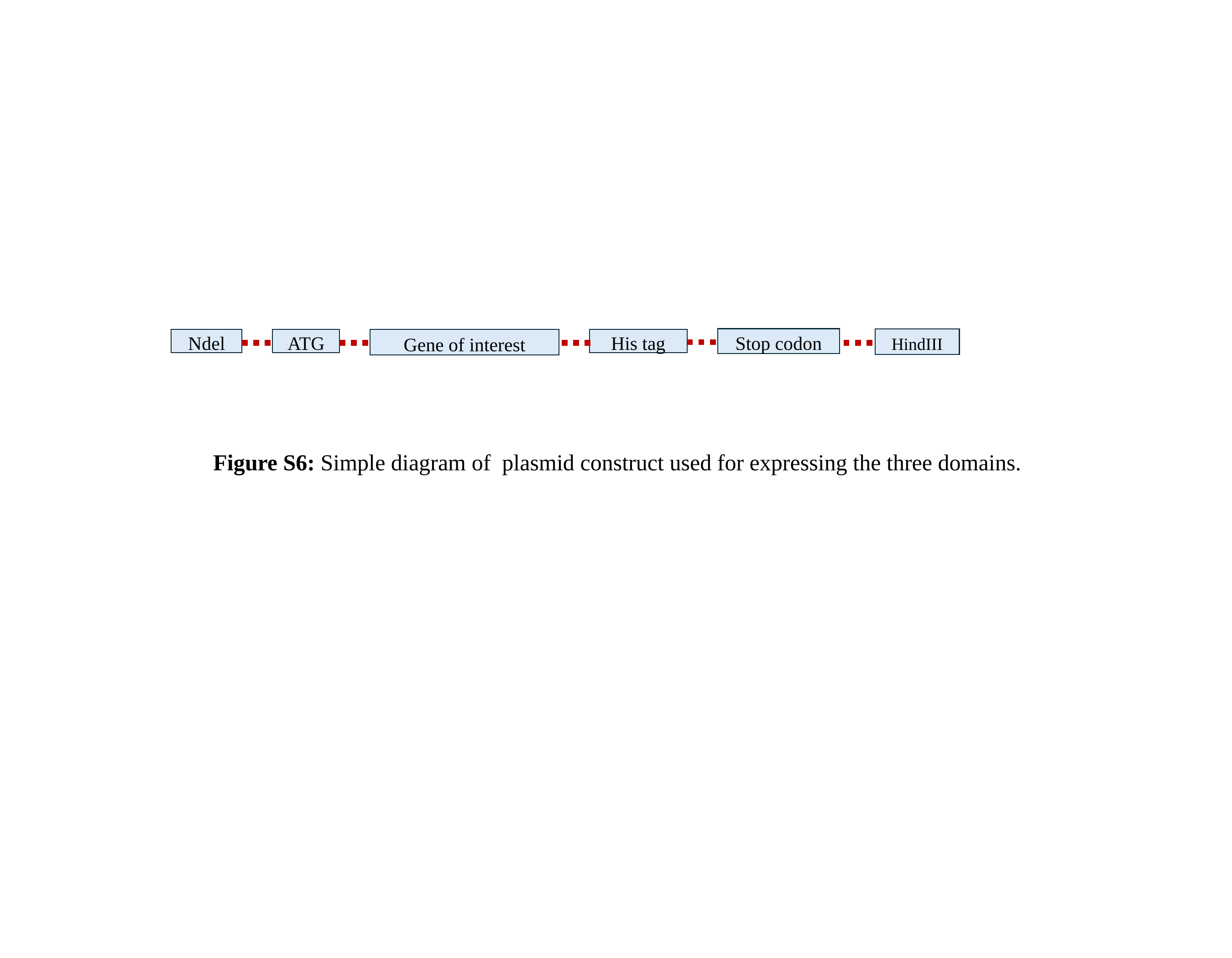

Stop codon
HindIII
Ndel
ATG
Gene of interest
His tag
Figure S6: Simple diagram of plasmid construct used for expressing the three domains.

## Slide 10
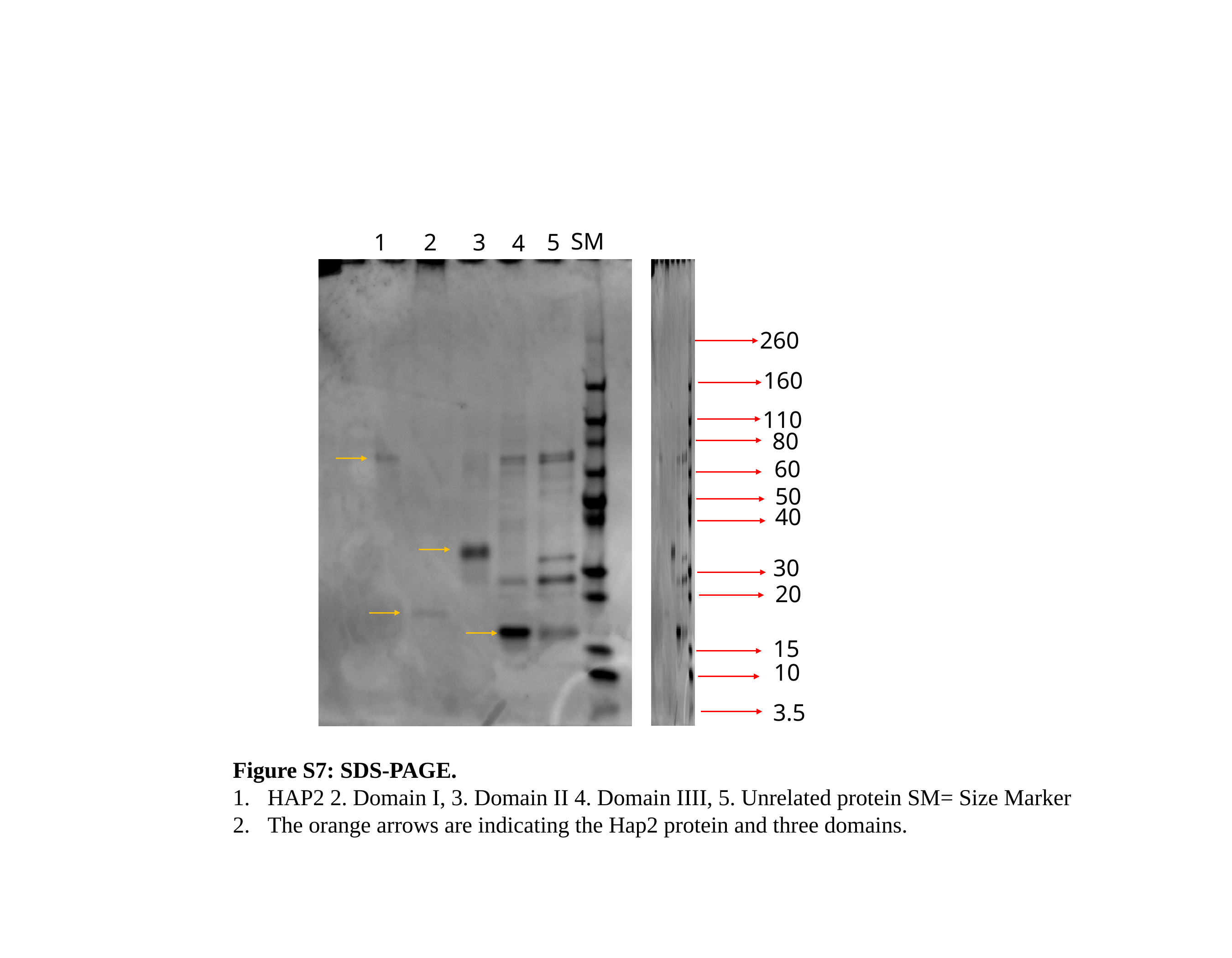

SM
1
2
3
5
4
260
160
110
80
60
50
40
30
20
15
10
3.5
Figure S7: SDS-PAGE.
HAP2 2. Domain I, 3. Domain II 4. Domain IIII, 5. Unrelated protein SM= Size Marker
The orange arrows are indicating the Hap2 protein and three domains.
